# Report and Remarks on a Series of Two Hundred Cataract Operations

**Published:** 1887-11

**Authors:** C. A. Bucklin

**Affiliations:** New York


### REPORT AND REMARKS ON A SERIES OF TWO HUNDRED CATARACT OPERATIONS.

By C. A. Bucklin, A. M., M. D., New York.

The first hundred of this series were cases which came under my personal observation while following the teachings of Hutchinson, Bowman, Cooper, Bader and Liebreich, of London; Mooren, of Dusseldorf; Rothmund, of Munich, and Arlt, Stellwag, Jæger and Mauthner, of Vienna. The second hundred I operated upon myself. I think the accident which occurred in the first hundred cases were quite as instructive to me as my own subsequent experience in operating.

Liebreich has had many flattering successes, but, on the other hand, he never could tell when he would have the most discouraging failure, from no other cause than the peculiarity of his operation. His corneal sloughing, above mentioned, fell later into Bader's hands, and he did what I never saw intentionally done before. Although the patient was seventy years of age, he made repeated discissions of the lens in the remaining eye, and after several months caused it to absorb sufficiently to give the man practical vision, a slight portion, however, of the nucleous remaining in the centre of the pupil.

The above experiment thoroughly demonstrated to me that it was practical to operate upon cataracts long before they were ripe.

In conversation with the late Dr. Frank Hamilton, he said, when the results of couching at the time of operation were satisfactory to him, they were usually, after a time, unsatisfactory to the patient; however, when he sometimes completely failed in displacing the cataract from the pupil, the results at a later time became permanently satisfactory to the patient. In the first instance he displaced the lens into the chamber of the vitreous, where it became a foreign body; in the second, his couching needle crushed through the lens, making an extensive discission. The lens, as in Bader's case, was gradually absorbed by the acqueous coming extensively in contact with its substance; in this way he certainly did obtain satisfactory and permanent visual improvement. Dr. Agnew, of this city, believes that the methods of Liebreich, owing to the prejudice of the profession against the man, never have received a fair trial, and he is giving them a trial.

Cocaine is excellent for all operations except enucleation, iridectomy and cataract ; I am fully convinced of its dangers in the two latter operations.

The German operators learned from experience that the debris left behind from attempting to remove a lens which had a clear cortical layer caused severe irritic reactions. They insisted on removing the iris sufficientlv to prevent its corners from becoming entangled in the wound. They tore more frequently than cut the anterior capsula. I think an operator in 1887, who would have had the temerity to remove a lens from the eye, through which fingers could still be counted at four feet, would have found poor support from his collegues had he been prosecuted for damages. Arlt, of Vienna, would wait for years till the layer of transparent cortex would become opaque. This was at that time the only way of avoiding the injurious effects from debris being left in the eye, thus causing iritic and irido-cystitic complications, and it cannot be denied that this was very sound practice.

The general introduetion of peripheral capsulotomy, for all cases of cataract extraction, by Dr. Knapp, certainly obviates, to a large extent, the objections to have a small amount of cortex remaining.

I have been continually experimenting, and now find no trouble in removing cataracts through which fingers can still be counted distinctly at four feet, and I am able to obtain a perfectly clear pupil without iritic complications.

My experience is, that out of one hundred cataract extractions ninety-six could count my fingers at from two to four feet. Two persons having cataract, who could not count fingers at any distance, lost their eyes from complications following cataract extractions. These failures were caused by accidents resulting from a hypermature condition of the cataract. I have carefully sought for the reason why experience thus far has brought me to a different conclusion from that arrived at by Dr. Noyes, whose experience certainly has been extensive. Under "Cystotomy," p. 246, I think I find an explanation of how our experience differs. He passes a cystotome below the pupillary margin a clean vertical cut in the anterior capsule. Now, I have made a horizonital peripheral cystotomy, corresponding as nearly as practical, to the upper margin of the lens ; occasionally, owing to an extra large lens, or a failure on my part to make the opening in the capsule large enough, the capsule has been torn in such a direction as to make an opening which was vertical, and extended below the pupillary margin. In every such case iritis followed, and what should be the movable margin of the iris became fastened to the capsule at the point of this vertical rupture.

The hundred cases which I have operated upon have taught me one lesson. With an empty stomach, profound etherization, absolute cleanliness, an iridectomy sufficiently broad to prevent any possibility of the corners of the iris becoming fast in the wound, and a clean peripheral cut in the capsule, the operation will not be complicated by any accident which is within the control of an operator. The wound will close in four days, without sufficient reaction for the patient to know from his sensations that the cataract has been removed ; extensive hemorrhage into the vitreous at the time of operation, or soon after, from the rupture of a diseased blood-vessel, is an unavoidable accident.

My results I sum up as as follows; *ninety-six could read the daily papers; two*, from disease existing behind the cataract, could not read, although the wound healed promptly without inflammatory reaction ; *two* were complete failures, from causes above detailed, and for which the unbiased scientist can in no way hold the operator or operation responsible.

I have not selected my cases, but have thus far operated upon cases presenting, irrespective of complications known to exist previous to the operation. The two cases where the light of a candle in a dark room could not be seen at a greater distance than ten feet, were very uncertain in locating the position of the light, and had been advised against an operation by very competent authority, owing to the probable complicated nature of the cataract. While I acknowledge the perfect soundness of the advice I cannot help calling attention to the instructiveness of the result, namely, vision sufficiently practical to read the daily papers. The prospective question in opthalmology will be, how shall we treat such cases of cataract where the disease is sufficiently advanced to prevent the individuals from following their usual vocation ? the prospects are not inviting where an attempt is made, after a preliminary iridectomy, to bring sufficient traumatism to bear upon the lens by applying the force through the cornea. Is it better to attempt to remove the cataractous lens at any stage, or to resort to some artificial means to ripen it ?

If the lens is to be ripened artificially, shall it be done by doing an iridectomy, and then introducing a de icate, polished instrument into the eye, stroking the anterior surface of the lens directly with its smooth surface ? or shall we dilate the pupil, and introduce into the eye a curved needle, the surface of its curve being a polished surface with which the lens may be carefully stroked ? or shall we carefully make a small discission of the lens for the purpose of ripening it ? my experience thus far enables me to say that they may still count fingers up to six feet before one is warranted in exposing his patients to the accidents which may follow any attempt to artificially ripen a cataract. It is better, when they cannot count fingers at a greater distance than six feet, to remove the lens and deal with any subsequent pupillary opacity as occasion requires, after all inflammatory reaction has subsided.

We must look to the experience of the future to decide which of the above methods is preferable.—*Medical Times.*

